# Resolution of taxonomic problems in Australian Harpalini, Abacetini, Pterostichini, and Oodini (Coleoptera, Carabidae)

**DOI:** 10.3897/zookeys.545.6752

**Published:** 2015-12-14

**Authors:** Kipling Will

**Affiliations:** 1 Essig Museum of Entomology, University of California, Berkeley, CA, USA

**Keywords:** Ground beetles, classification, Australia, New Zealand

## Abstract

Taxonomic changes are made for several problematic Australian Carabidae in the tribes Harpalini, Abacetini, Pterostichini, and Oodini. Examination of types resulted in the synonymy of *Veradia* Castelnau, 1867 with *Leconomerus* Chaudoir, 1850; *Nelidus* Chaudoir, 1878, *Feronista* Moore, 1965, and *Australomasoreus* Baehr, 2007 with *Cerabilia* Castelnau, 1867; and newly combining *Fouquetius variabilis* Straneo, 1960 in the genus *Pediomorphus* Chaudoir, 1878; *Australomasoreus monteithi* Baehr, 2007 in the genus *Cerabilia* Castelnau, 1867; and *Anatrichis lilliputana* W.J. Macleay, 1888 in the genus *Nanodiodes* Bousquet, 1996. *Cuneipectus* Sloane, 1907 is placed in Pterostichini Bonelli, 1810, which is a senior synonym of Cuneipectini Sloane, 1907.

## Introduction

In a continuing effort to make the faunal list of Australian carabid beetles as accurate as possible, I sought out and studied type specimens for a number of historically problematic taxa. Results of my study require a number of adjustments to recognized taxa.

## Methods

Institution codens used here for material examined: Australian National Insect Collection (ANIC) CSIRO, Canberra; Essig Museum of Entomology (EMEC), Berkeley; Museo Civico di Storia Naturale “Giacomo Doria” (MCSN), Genova; Muséum National d'Histoire Naturelle, (MNHN), Paris; Museum of Comparative Zoology (MCZ), Harvard; Naturhistorisches Museum Basel (NMB), Switzerland; Queensland Museum (QM), Brisbane; and Western Australian Museum (WAM), Perth.

## Results and Discussion

### Harpalini

Taxon classificationAnimaliaColeopteraCarabidae

Bonelli, 1810

Lecanomerus Chaudoir, 1850; type species, *Lecanomerus insidiosus* Chaudoir, 1850.
Veradia Castelnau, F.L. Laporte de, 1867; type species *Veradia brisbanensis* Castelnau, F.L. Laporte de, 1867. **syn. n.**
Lecanomerus brisbanensis (Castelnau, 1867). **comb. n.**

#### Material examined.

Holotype, male [MCSN]. Type locality Brisbane. A female specimen, “26.49S 151.58E [29°49'S / 151°58'E] Yarraman QLD State F. No. 282, 31 Mar. 1982, R.A. Barrett, M. Lenz, L. Miller”//”Rotten log” [ANIC].

#### Notes.

Originally this species was placed by Castelnau (1867) near *Moriodema* Castelnau, 1867, a Moriomorphini taxon, which was then considered to be within Pterostichini. Subsequently it was moved to Harpalini by Chaudoir (1880) and according to Chaudoir it did not differ from *Hypharpax* W.S. Macleay, 1825. Sloane (1898) agreed with the placement in Harpalini, but deferred on the generic assignment and its possible similarity to *Hypharpax*. Straneo (1941) thoroughly reviewed the pertinent literature and studied the type specimen of *Veradia brisbanensis*. He concurred with the placement in Harpalini and suggested there were similarities with *Nemaglossa* Sloane, 1920 (=*Lecanomerus* Chaudoir, 1850, not *Nemaglossa* Solier, 1849), *Euthenarus* Bates, 1874 and *Diaphoromerus* Chaudoir, 1843 (= Notiobia (Anisotarsus) Chaudoir, 1843). These taxa fall in three different tribes of Harpalini and Straneo pointed out that without access to Australian material for comparison that he could not make a decision regarding the status or relationships of the genus and species. Moore et al. (1987) maintained the genus in Harpalitae incertae sedis, accurately reflecting the uncertainty of the placement of the taxon at that time.

I examined the holotype and confirm that it has typical Harpalini character states, e.g. single supraorbital seta and no elytral plica, and does not have any character states that would place it in any other tribe. Additionally the male has the front and middle tarsomeres expanded with spongy ventral pads, the penultimate labial palpomere is bisetose, the posterior lateromarginal seta of pronotum is absent and the angular base of stria 1 is absent. This combination of character states is consistent with placement of this taxon in subtribe Pelmatellina and is identical to the state combination found in many Australia *Lecanomerus* species. Based on this evidence, *Veradia* is considered a junior synonym of *Lecanomerus*.

A search in the holdings of the ANIC and QM did not yield any additional specimens of this species beyond the single female, but at least six very similar looking *Lecanomerus* species were found. Each was distinctly different, but all are very likely closely related based on their general similarity. How many of these are currently named species cannot be assessed without recourse to the types.

### Abacetini Chaudoir, 1873

#### Pediomorphus variabilis

Taxon classificationAnimaliaColeopteraCarabidae

(Straneo, 1960) comb. n.

Fouquetius variabilis Straneo, 1960

##### Material examined.

Holotype, male [NMB]. Type locality Katherine, Northern Territory. Examined images only.

##### Notes.

Straneo (1960) discusses at length his sense that *Holconotus* Schmidt-Goebel, 1846 (= *Fouquetius* Maindron, 1906) and *Pediomoprhus* Chaudoir, 1878 are closely related and that *Pediomorphus macleayi* Sloane, 1900 could be a species of *Holconotus*. Moore (1965) confirmed that *Pediomorphus macleayi* is a true *Pediomorphus*. Straneo's conclusions are based on very limited material and he did not discuss characters that allow for clear placement of species in these two genera. Among other characteristics, *Pediomorphus* has distinctly expanded penultimate labial palpomeres not found in *Holconotus*, while the elytral lateral bead is distinctly, finely serrate in *Holconotus* and smooth in *Pediomorphus*. The type specimen of *Pediomorphus variabilis* has clearly expanded penultimate labial palpomeres and smooth elytral lateral beads. Given the new combination, *Holconotus* is removed from the Australian faunal list.

#### Cerabilia

Taxon classificationAnimaliaColeopteraCarabidae

Castelnau, 1867

Cerabilia Castelnau, 1867; type species, *Cerabilia maori*, Castelnau, F.L. Laporte de, 1867.
Zabronothus Broun 1893; type species, *Zabronothus striatulus* Broun, 1893.
Nelidus Chaudoir, 1878; type species, *Nelidus australis* Chaudoir, 1878. **syn. n.**
Australomasoreus Baehr, 2007; type species, *Australomasoreus monteithi* Baehr, 2007. **syn. n.**
Feronista Moore, 1965; type species, *Feronista amaroides* Moore, 1965. **syn. n.**

#### Cerabilia australis

Taxon classificationAnimaliaColeopteraCarabidae

(Chaudoir, 1878) comb. n.

Nelidus australis Chaudoir, 1878

##### Material examined.

Holotype, male [MNHN], type locality given as Paroo River area (QLD or NSW), but probably erroneous. See below.

#### Cerabilia monteithi

Taxon classificationAnimaliaColeopteraCarabidae

(Baehr, 2007) comb. n.

Australomasoreus monteithi Baehr, 2007

##### Material examined.

Holotype, male [QM]. Type locality Bulburin State Forest via Many Peaks, Qld. An additional 12 specimens from the type locality [EMEC, QM].

##### Notes.

*Cerabilia*, sensu Will (2011) includes Australian species placed in *Feronista* by Moore et al (1987) and *Cerabilia* species from New Zealand and New Caledonia. Baehr (2007) described *Australomasoreus monteithi* as a Masoreini, but he clearly noted that this placement was both anomalous for the species' characteristics and biogeography. Study of the type and additional material for both morphology and DNA data (Will unpubl.) clearly places this species in *Cerabilia*.

*Cerabilia australis* is known only from the holotype specimen and was reported as coming from the Paroo River area. However, this specimen is unlike any Australian species of carabid and is very similar to *Cerabilia* species from New Zealand. It may in fact be a synonym of one of the described New Zealand species, but until their types are studied this cannot be established. The Australian *Cerabilia* species are all restricted to the higher elevation rainforests in the northeastern coastal region. The Paroo River runs through the semi-arid inland region of southwestern Queensland and northwestern New South Wales and is both geographically and environmentally distant from any location where *Cerabilia* has been found in Australia. Likely the type locality was erroneously reported.

### Pterostichini

Taxon classificationAnimaliaColeopteraCarabidae

Bonelli, 1810

Cuneipectini Sloane, 1907. Syn. n.
Cuneipectus Sloane, 1907; type species, *Cuneipectus frenchi* Sloane, 1907.

#### Material examined.

Holotype, *Cuneipectus frenchi* [ANIC] and three additional specimens [ANIC, MCZ]; ten specimens of *Cuniepectus foveatus* Sloane, 1915 [EMEC].

#### Notes.

Sloane described a new tribe for *Cuneipectus* suggesting that it belonged “at the beginning of the Trigonotomid series of the subfamily Harpalinae”, i.e. as sister to a group Pterostichini. Subsequent authors have placed it between Harpalini and Chlaeniini (Csiki 1931), near chaetogenyines, chlaeniines, oodines, and licinines (Callistitae sensu Erwin and Sims (1984) and Erwin (1985, 1991)) in Licininae (Lorenz 2005) in Pterostichitae (Moore et al. 1987) or Pterostichini (Lawrence and Slipinski 2013). Moore (1965) did not include *Cuneipectus* in his treatment of Australian Pterostichinae. Aside from the original description, there has not been a discussion of the characteristics of *Cuneipectus*. Its variable placement, non-inclusion in Moore's (1965) treatment and frequent association with Chlaeniini and Licinini by various authors apparently stems from the species being described as having a single supraorbital setae in combination with the presence of an elytral plica. However, supraorbital seta number is variable, with some individuals having one and others two above each eye. Other characteristics are typical of Australian Pterostichini, including the presence of the spermathecal gland duct diverticulum (sgd) in the female (Liebherr and Will 1998). The sgd is typical in many pterostichines including Australian taxa like *Prosopogmus* Chaudoir, 1865 (Will 2011), *Paranurus* Tshitshérine, 1901 (Liebherr and Will 1998) and *Trichosternus* Chaudoir, 1865 (Will unpubl.). The sgd is not known to be present in any Chlaeniini or Licinini. Additionally, preliminary analyses of DNA data (Will unpubl.) consistently places *Cuneipectus* with Australian Pterostichini. Based on this evidence, *Cuneipectus* is placed in Pterostichini and Cuneipectini is synonymized.

### Oodini

Taxon classificationAnimaliaColeopteraCarabidae

LaFerté-Sénectère, 1851

Nanodiodes lilliputana (W.J. Macleay, 1888)
Anatrichis lilliputana W.J. Macleay, 1888

#### Material Examined.

Syntypes [ANIC], type locality, King Sound, Western Australia. Additional material in ANIC and WAM examined.

#### Notes.

*Nanodiodes* Bousquet, 1996 was proposed by Bousquet (1996) to replace *Nanodes* Habu, 1956 and he moved all species that where included by Moore et al. (1987) in *Anatrichus* LeConte, 1853 into this genus except for *Anatrichis lilliputana*, which Bousquet had not studied. Although some subsequent catalogs (e.g., Lorenz 2005) treated this species as *Nanodiodes lilliputana*, there is no indication that the character states were confirmed. I examined the syntypes and found the following: submentum with pairs of setae at the lateral edge; mesocoxa with a posterior seta and; metatrochanter without a seta. This combination is consistent with *Nanodiodes*, confirming that it shares the putative synapomorphic character states with species currently included in that genus. *Anatrichis* is therefore not found in the Australian fauna.

## Supplementary Material

XML Treatment for Harpalini

XML Treatment for Pediomorphus variabilis

XML Treatment for Cerabilia

XML Treatment for Cerabilia australis

XML Treatment for Cerabilia monteithi

XML Treatment for Pterostichini

XML Treatment for Oodini
